# Large Scale Manufacturing of Wharton's Jelly Mesenchymal Stromal Cells for Clinical Use

**DOI:** 10.1155/sci/5167739

**Published:** 2025-10-31

**Authors:** Rupal P. Soder, David Splan, Nathaniel R. Dudley, Mark Szczypka, Sunil Abhyankar

**Affiliations:** ^1^Midwest Stem Cell Therapy Center, University of Kansas Medical Center, Kansas City, Kansas, USA; ^2^Sartorius Stedim North America Inc., Ann Arbor, Michigan, USA; ^3^Hematological Malignancies and Cellular Therapy, University of Kansas Cancer Center, Kansas City, Kansas, USA

**Keywords:** microcarriers, stirred-tank bioreactor, three-dimensional suspension culture, Wharton's jelly mesenchymal stromal cell

## Abstract

Therapies utilizing human mesenchymal stromal cells (MSCs) are advancing through clinical trials, emphasizing the need for reliable, scalable, and cost-efficient manufacturing processes to support the lot sizes necessary for commercial-scale production. Wharton's jelly MSCs (WJMSCs) are valued for their regenerative abilities and immunomodulatory and anti-inflammatory properties, which contribute to tissue repair. With growing therapeutic demand, the production of WJMSCs must scale to yield billions of cells while maintaining their essential characteristics—identity, purity, and potency—necessary for clinical and regulatory compliance. Achieving such magnitude of expansion entails the utilization of current good manufacturing practice (cGMP)-compliant scalable culture systems that allow bioprocess control and monitoring. This study aimed to establish a scalable serum-/xeno-free expansion process representing a critical step towards a cGMP-compliant large-scale production platform for WJMSC-based clinical applications. Using our in-house GMP-manufactured WJMSCs, which were tested in a Phase Ib clinical trial (NCT03158896), we have previously optimized various culture parameters using a microcarrier (MC)-based three-dimensional (3D) culture system in spinner flasks and demonstrated successful WJMSC expansion. In the present study, we successfully translated culture conditions to a 2 L followed by a STR50 (50 L) stirred-tank bioreactor (BR) (STR), adhering to cGMP requirements. The culture system in the 2 and 50 LBRs supported cell concentrations of approximately 1.2 x 10^6^ cells/mL and attained 24-fold and 27-fold expansion, respectively, with a yield of approximately 37 billion cells in the 50 L culture system after 7 days with a 95% harvest efficiency. Following expansion, WJMSCs preserved their characteristic phenotypes, differentiation potential, chromosomal stability, functional capabilities, and sterility across all tested culture systems. We conclude that the large-scale expansion process of WJMSCs in the STR described herein is highly adaptable to the scale necessary to fulfill the commercial demand for high quality clinical-grade MSCs.

## 1. Introduction

Mesenchymal stromal cells (MSCs) have emerged as a prominent focus in biomedical research due to their favorable safety profile in human studies and, more recently, their therapeutic promise as evidenced by outcomes from advanced-phase clinical trials [1–4]. Among the various MSC sources, those derived from Wharton's jelly of the human umbilical cord are especially notable. Their unique characteristics—including minimal immunogenicity, high proliferation capacity, immune modulation, and regenerative potential—make them ideal candidates for ongoing clinical investigations. These cells are currently being investigated in a broad spectrum of clinical trials targeting conditions such as immune-mediated diseases, musculoskeletal injuries, cardiovascular dysfunctions, liver disorders, and neurodegenerative diseases [5–8]. The escalating interest in Wharton's jelly-derived MSCs (WJMSCs) for therapeutic applications has led to a significant increase in demand for large-scale and clinical-grade cell production. Therapeutic dosing requirements can vary widely; for instance, treatments may necessitate between 1 and 10 x 0^6^ cells per kilogram of patient body weight, translating to approximately 10^8^–10^9^ cells for a 100 kg individual. In the context of high-throughput drug screening or engineered tissue platforms, the number of cells required can exceed 10 × 10^9^ per campaign—a scale far beyond what planar systems can efficiently deliver. While traditional two-dimensional (2D) culture systems can accommodate small-scale production, they fall short when it comes to meeting the needs of extensive clinical trials or large patient populations. To address these challenges, the field is increasingly turning to three-dimensional (3D) culture systems, such as microcarrier (MC)-based bioreactors (BRs), which offer enhanced scalability and efficiency [9].

Compared with 2D stacked multilayer vessels commonly used for scaling up expansion of several anchorage-dependent cells, 3D MC systems provide a significantly increased surface-to-volume ratio maximizing space efficiency in a controlled homogenous microenvironment [10]. One of the first demonstrations of the successful expansion of MSCs on MCs was reported in 2007 [11]. Multiple studies have since confirmed the feasibility of expansion of MSCs on MCs within fully controllable dynamic BR systems [12–15]. MC-based BR systems provide advantages for cell culture such as uniform culture conditions throughout, via impeller agitation, precise process control, adaptable operation modes, accessible sampling, and in situ harvest capabilities. Efforts to expand WJMSCs in regulated stirred-tank BRs (STRs) have mostly been limited to volumes ranging from 800 mL to 3 L [16–21] with a recent study describing a GMP-compliant expansion of WJMSC using MC and 3L single-use STR [17]. Pilot scale investigations involving volumes between 10 and 100 L are essential due to the substantial cell quantities needed for therapeutic applications and to ensure a reliable procedure. While insights into the 3D WJMSC expansion process on a smaller scale have been reported, it remains unclear whether a systemic approach was employed to determine the most suitable culture conditions for WJMSC expansion in a STR. Moreover, the seed train process development and expansion of WJMSCs on a larger scale STR (50 L) combined with evaluation of critical quality attributes of clinical grade 3D expanded WJMSCs have not been reported to date.

The present study aimed to optimize and establish a MC-based 3D culture process in an STR system to expand clinical-grade WJMSCs and evaluate the functional characteristics of expanded cells. We also sought to verify that the expanded cells met all the acceptance criteria established for our ongoing Phase Ib clinical trial in aGvHD (NCT03158896).

As previously reported, we systematically optimized a MC-based culture process using a stepwise approach that began with 2D static cultures for MC screening (biocompatibility assessment), followed by 3D dynamic culture in small-scale spinner flasks to refine key parameters such as seeding density, agitation strategy, and feeding regimen [22]. Building upon this small-scale foundation, and with the objective of manufacturing large quantities of clinical-grade WJMSCs, we translated the optimized process to a GMP-compliant large-scale STR system. This scale-up required further additional refinement—optimization of critical parameters, including impeller speed, dissolved oxygen (DO) setpoints, and the control strategies—to accommodate the larger vessel dynamics and meet the metabolic demands of the culture. This process utilized a scalable seed train incorporating the Sartorius Univessel 2 L glass and Biostat STR 50 L (STR50; 30 L working volume) single-use BR systems, allowing for robust and controlled expansion of WJMSCs under conditions suitable for clinical production. Following a 13-day culture spanning the 2 L and STR50 BRs, approximately 37 billion cells were generated with 95% harvest efficiency. The harvested WJMSCs maintained cell viability, phenotypic characteristics, cytogenetic profile, multipotency, and strong immunosuppressive potential. Importantly, these assessments aligned with the acceptance criteria established for our ongoing Phase Ib clinical trial demonstrating large scale manufacturing of clinical-grade cells while retaining their essential therapeutic properties.

## 2. Materials and Methods

### 2.1. WJMSC Clinical Grade Culture Expansion Process

The University of Kansas Medical Center (KUMC) Institutional Review Board (IRB) approved the study (Study IRB# 00001546), and informed consent was obtained from mothers before umbilical cord collection. WJMSCs were isolated from umbilical cords (typically 15–20 cm long) collected from healthy full-term women (18–35 years old) who underwent elective cesarean section. To qualify as cord donors, mothers were tested and shown to be free of human immunodeficiency virus (HIV) Types 1 and 2, hepatitis A, B, and C, *Treponema pallidum*, *Chlamydia trachomatis*, *Neisseria gonorrheae*, and HTLV 1 and 2. WJMSCs product was manufactured by the Midwest Stem Cell Therapy Center (MSCTC) at KUMC under IND 017672. Briefly, fresh human umbilical cords were rinsed twice with phosphate-buffered saline supplemented with antibiotic-antimycotic to remove traces of unwanted blood cell contaminants. The two arteries and one vein were removed and the cleaned cored sections were then minced into 1–2 mm explants and placed in cell culture dishes containing complete medium to start WJMSC explantation and expansion for traditional 2D flask culture. Half of the medium was replaced every 3 days, and well-developed colonies appeared after 10 days. Early-passage WJMSCs (passages 2–3) were used for downstream experiments. Following isolation, WJMSCs were cultured in xeno-free and serum-free culture media (MSC Nutristem XF Basal Medium (Sartorius, Cat#05-200-1 A) supplemented with 0.06% MSC NutriStem XF Supplement Mix (Cat#05-201-1U) and 5% PLTGold Human Platelet Lysate, (PLT Gold Clinical Grade, Cat#PLTGOLD500GMP), designated as “complete medium” and expanded (until passage 5) under current good manufacturing practice (cGMP)/good tissue practice (cGTP) (cGMP/cGTP) standards. Cells were then cryopreserved using plasmalyte (Baxter, Cat#2B2543), 5% HSA (Shire, Cat#NDC44206-251-05) and 10% DMSO (Origin Biomedical, Cat#CP-50) and held on ice until transfer to a controlled rate freezing device and finally were moved to the vapor phase of liquid nitrogen for long-term storage.

### 2.2. WJMSC Expansion in Bioreactors

A process for expansion of WJMSCs was developed using commercially available and cGMP compatible, Biostat STR BRs and the Biobrain automation platform. Specifically, a Biostat B 2 L Univessel glass (2 L) BR and a Biostat B STR 50L Generation 3 single-use bioreactor (STR50; 50 L) were used in this study (Sartorius). A summary of the process flow with operating parameters and BR set points for the run are shown in Table 1. Clinical grade WJMSCs from passage 2 were thawed from frozen stocks, pelleted, and washed to remove the cryopreservation medium for cell expansion. Cells were counted and resuspended in a complete culture medium and seeded into a CellStack 5 tissue culture flask (CS5, VWR, Cat#66025-656) at 4500 cells/cm^2^. Cells were expanded for 4 days in planar culture and harvest was performed using 0.04 mL/cm^2^ 0.5x TrypLE (in 1 × PBS) at 37°C for 10 min following which enzyme activity was quenched with complete medium. Cells were counted and the suspension was used to seed onto SoloHill Collagen coated MCs (Sartorius, Cat#CIR-221-020) in a Biostat 2 L BR containing supplemented Nutristem XF medium with PLT Gold at a MC density of 10 cm^2^/mL (28 g/L). During the first 4 h of culture, deemed the “attachment phase," concentrations of supplement mix and PLT in the cell culture medium were held to 0.05% of their final concentrations. Our unpublished observation indicated that proteins found in supplements such as albumin in PLT, can inhibit cell attachment, and that reducing the concentrations to very low levels during the first few hours of culture can improve attachment. An additional measure employed to aid in cell attachment included an intermittent agitation profile, which consisted of cycling between brief periods of agitation (3 min), followed by periods without agitation (30 min). This cycling was repeated for a total of eight cycles, at which time the supplements were brought to their normal levels and agitation was switched from intermittent to continuous mode. Samples were collected at approximately 24 h postinoculation to evaluate cell attachment to the MC population. Samples were fixed with 4% formaldehyde and labeled with 4′,6-diamidino-2-phenylindole (DAPI) (VWR, Cat#422801-BL), a DNA-binding fluorescent dye. Fluorescence microscopy using a Nikon TiE microscope and NIS Elements software enabled visualization and quantification of nuclei attached to the MCs.

Daily samples were retrieved from the 2 L BR to monitor cell growth and nutrient and metabolite levels. After 6 days of culture, cells were harvested from the MCs, following PBS washes and trypsinization. Briefly, cells were harvested from MCs by incubation with 0.5 × TrypLE Select (in 1x PBS) at 37°C for 30 min with gentle agitation. To quench the enzyme reaction and collect cells, a volume of complete medium equal to that for TrypLE was added to the BR originally containing the cells and beads. After enzymatic dissociation, the cell–MC mixture was filtered through a 70 μm mesh to separate the liberated cells from the MCs. The strainer was rinsed with culture medium to maximize cell recovery into the collection vessel. Cells were quantified and used to seed the STR50 (50 L) BR at a working volume of 30 L. Cell concentration of the suspension was determined, and the cell feed was used to seed onto collagen-coated MCs in a STR50 containing complete culture medium with PLT Gold medium and a MC density of 10 cm^2^/mL (28 g/L). As with the Biostat 2 L BR, a reduced supplement (0.05%) and an intermittent agitation attachment phase were employed. Samples were collected to evaluate attachment and were processed similarly as with the Biostat 2 L Univessel. Daily samples were retrieved from the STR50 BR to monitor cell growth and nutrient and metabolite levels. After 7 days of culture, cells were harvested from MCs following PBS washes and trypsinization quantified, and cryopreserved in liquid nitrogen.

### 2.3. Cell Growth and Viability Assessment

Representative samples were collected from the cell suspension obtained from planar cell culture vessels and BRs, and cell counts were performed using a Nucleocounter NC-200. To perform cell enumeration and determine viability, samples were loaded into a Via1-Cassette (ChemoMetec, Cat#941-0012) containing the fluorophores acridine orange and DAPI and cell numbers and viability were measured by the Nucleocounter NC-200. Retained MCs from BR samples were dried and weighed to determine surface area and to calculate cells per cm^2^ for each sample. Harvest efficiency was determined by comparing cell yields from a representative small-scale harvest to those from the full-scale BR harvest. Prior to full harvest, a 100–200 mL sample was withdrawn and processed using a conventional wash and enzymatic detachment protocol, which was defined as the baseline (100% recovery). At the end of culture, the entire BR was harvested using the optimized enzymatic dissociation protocol developed at small scale to generate single-cell suspensions from MCs. The liberated cells were then passed through a 70 μm mesh to physically separate them from MCs, and the resulting suspension was sampled for enumeration. Cell number and viability were measured using the Nucleocounter NC-200, with replicate counts performed on independent aliquots. To correct for sampling variability, MCs were dried and weighed at both scales to normalize for total available surface area. Harvest efficiency was calculated as the ratio of cells recovered in the full-scale harvest relative to the extrapolated baseline from the small-scale sample, corrected for surface area.

### 2.4. Nutrient/Metabolite Analysis

Nutrient consumption and metabolite production were analyzed throughout the cultures at specific time points. Daily samples retrieved from BRs were centrifuged at 200 × *g* for 5 min at room temperature to pellet any cells and 1 mL of supernatant was analyzed using a Cedex Bio HT Analyzer. Consumption of glucose, glutamine, production of lactate, and ammonia accumulation were measured. Results are shown up to day 5 for the Biostat 2 L Univessel and up to day 7 for the STR50.

### 2.5. Characterization of WJMSC Using Flow Cytometry

Phenotypic characterization for each WJMSC product harvested after expansion from 2D and 3D cultures was performed. The percentages of cells positive for markers characteristic of MSCs (CD73, CD105, and CD90) and negative for hematopoietic, macrophage, and B cell markers (HLA-DR, CD19, CD45, CD11b and CD34) were determined on total cells. Cells were characterized by flow cytometry using the BD Stem flow Human MSC analysis kit (BD Biosciences, San Diego, CA) and were evaluated using Becton Dickenson LSR II flow cytometer. Data were analyzed using FACSDiva software.

### 2.6. Multilineage Differentiation

WJMSCs were directed toward adipogenic, chondrogenic, and osteogenic lineages by substituting the standard expansion medium with lineage-specific differentiation media (StemPro kits, Life Technologies: Cat#A10070-01 for adipogenesis, A10071-01 for chondrogenesis, and A10072-01 for osteogenesis), as per the manufacturer's instructions. After a 21-day induction period, cells were gently washed with PBS, fixed in 4% paraformaldehyde for 10 min, and subsequently stained to confirm lineage-specific differentiation using oil red O (adipocytes), toluidine blue (chondrocytes), and alizarin red (osteocytes).

### 2.7. Immunosuppression

Immunosuppressive potency of WJMSCs was determined by evaluating the ability to inhibit phytohemagglutinin (PHA)-induced proliferation of human peripheral blood mononuclear cells (PBMCs) in coculture. A known number of WJMSCs were seeded in 96-well plates and after 24 h, 10 μg/mL mitomycin-C was added to inhibit cell proliferation. The treated cells were incubated for 2 h at 37°C and then washed five times with culture medium. A known number of PBMCs were added to each well, stimulated with PHA to activate T-cell proliferation and cocultured for 3 days. Inhibition was determined by flow cytometry analysis of T-cell proliferation, compared to a WJMSC-free culture. The effect of WJMSCs on PBMC stimulation response was calculated as percentage suppression compared with the proliferative response in the positive control without WJMSCs (± standard deviation of the mean). The positive control was set to 0% suppression.

### 2.8. Cytogenetics

Chromosomal analysis of the final WJMSC product was performed by the Cytogenetics Laboratory within the Division of Genomic Diagnostics at KUMC. Cells were harvested, then fixed using a 3:1 methanol-to-acetic acid solution. The fixed cell suspensions were applied to glass slides and subjected to GTL banding (Giemsa/trypsin/Leishman stain). Metaphase spreads were examined microscopically and compared to internal reference standards to assess karyotypic stability.

### 2.9. Microbiology Testing

Endotoxin quantification was performed with the Endosafe-PTS system (Charles River Laboratories, Wilmington, MA, USA) following the manufacturer's instructions. MSC sterility was assessed by bacterial culture (aerobic and anaerobic) and fungal culture for 14 days and detection of mycoplasma was conducted by luminometry per manufacturer's instructions (Mycoalert, Lonza BioScience, Verviers, Belgium).

### 2.10. Statistical Analysis

Data were compiled and analyzed using Microsoft Excel (Microsoft Corp., Redmond, WA, USA). Values are reported as the mean ± standard deviation. Statistical comparisons between two independent groups were conducted using unpaired, two-tailed Student's *t*-tests, with Welch's correction applied in cases of unequal variances. Statistical significance was defined as a *p*-value less than 0.05 (*⁣*^*∗*^).

## 3. Results

Studies were undertaken to assess the large-scale manufacturing of WJMSCs on collagen-coated MCs in 2 and 50 L STRs under optimized harvest and seeding conditions. Cell attachment, growth kinetics, metabolite profiles, and postexpansion quality attributes were systematically evaluated.

### 3.1. Culture in Bioreactors

#### 3.1.1. Cell Attachment to MCs

**Figure 1:**
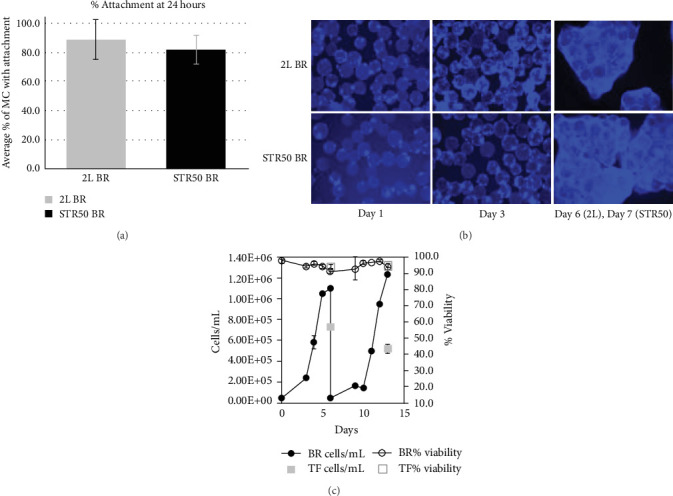
Cell attachment to microcarriers. (A) Attachment of WJMSC to microcarriers (MCs) at 24-h timepoint in 2 L and STR50 bioreactors (BRs) using a seeding density of 4500 cells/cm^2^ and following a reduced supplement and intermittent agitation strategy (*n* = 3). (B) Images of DAPI-stained cells on microcarriers demonstrate cell attachment and growth in bioreactors, shown for days 1, 3, and 6 for Biostat 2 L and days 1, 3, and 7 for STR50 (*n* = 3). (C) Excellent growth and viability of WJMSCs observed on microcarriers in the 2 L and STR50 bioreactors (BRs), exceeding that of the T-flask (TF) controls. Cell counts and viability at days 3–6 for the Biostat 2 L and at days 3–7 for the STR50. T-flask control cell count and viability are shown at day 6 for 2 L and day 7 for STR50. Data are presented as mean ± standard deviation (*n* = 3).

One of the first requirements for successful suspension culture of adherent cells on MCs is to achieve efficient attachment of cells to the MC population. Using harvest conditions optimized at a small scale, robust and single-cell suspensions were seeded into the Biostat 2 L and STR50 BRs, each at a seeding density of 4500 cells/cm^2^ (Table 2). Following the reduced supplement and intermittent agitation strategy, samples were retrieved at 24 h to assess the attachment of cells on collagen-coated MCs. In this study, the average percentage of MCs with cell attachment exceeded 80% at the 24-h timepoint for both Biostat 2 L and STR50 BRs (Figure 1A). Immunofluorescence demonstrated expansion of cells on MCs from both BRs over their respective culture periods (Figure 1B).

### 3.2. Cell Growth and Harvest

Figure 1C shows the growth of cells from the BRs and T-flask (TF)controls throughout the culture. Samples were collected on days 3–6 for the Biostat 2 L and days 3–7 for the STR50. TF control cell counts reached 7.3x10^5^ cells/mL by day 6 for the 2D-controls in the 2 L run and 5.3 x 10^5^ cells/mL for the 2D-controls in the STR50 run. The BR conditions reached 1.1 x 10^6^ cells/mL by day 6 and 1.2x10^6^ cells/mL by day 7, for the 2 L and STR50, respectively. Viability was greater than 90% for all conditions. Cell counts in cells/mL from the cultures are shown in Figure 1C to demonstrate one of the advantages MC cultures have over planar (TF) cultures. Unlike planar cultures, MC systems permit scalable surface area expansion by simply increasing bead concentration. This flexibility supports significantly higher cell densities within the same culture volume, enhancing volumetric productivity. At a MC density of 10 cm^2^/mL, the BR samples reached significantly higher cell densities compared to the planar culture counterparts which are typically limited to a 5 cm^2^/mL surface area-to-volume ratio due to gas exchange requirements. As demonstrated in Figure 1B aggregates of cells and MCs began to form as the culture progressed, allowing cells to densely populate virtually the entire population of MCs, with aggregate ranging from 2 to 50+ MCs.

### 3.3. Final Harvest Results for the Expansion Process

Table 1 shows the expansion process results, which proceeded from 2D flasks to the STR50 BR. The starting number of cells required to seed the CellSTACK5 was 1.4 x 10^7^. This number was expanded for 4 days, generating enough cells (1.4 x 10^8^ cells) to seed the Biostat 2 L BR. Following a 6-day expansion in the STR, a harvest density of 1.1 x 10^6^ cells/mL was reached, equating to a total of 2.2 x 10^9^ cells, or a 24.4-fold expansion in the Biostat 2 L BR. A total of 1.35 x 10^9^ cells of this harvested suspension was used to seed the STR50 at 30 L. Following the 7-day expansion in the single-use STR50 BR, a harvest density of 1.24 x 10^6^ cells/mL was reached, equating to 37.2 x 10^9^ cells, or a 27.6-fold expansion in the STR50 BR. Over the entire 17-day culture period, a 2657-fold increase was achieved.

### 3.4. Nutrient and Metabolite Profile

**Figure 2:**
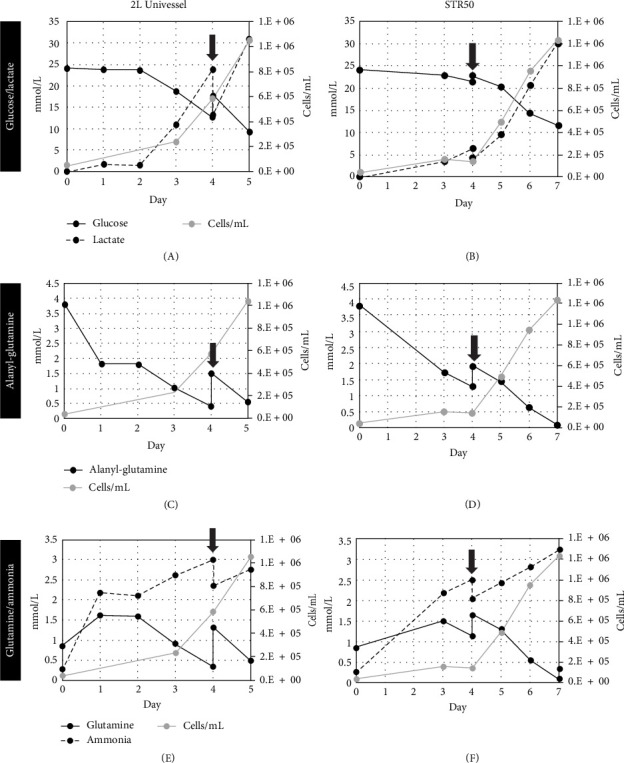
Metabolic profile for the Biostat 2 L Univessel and the STR50. (A–B) Glucose consumption and lactate production increase as WJMSC growth progresses for both the 2L Univessel and the STR50. (C–D) 2 L Univessel and STR50 bioreactors both show decreasing levels of alanyl-glutamine with increasing WJMSC growth. (E–F) As WJMSC growth progresses for both the 2 L Univessel and the STR50, ultimate levels for glutamine consumption and ammonia production increase. Results shown are up to day 5 for the 2 L Univessel and up to day 7 for the STR50. Values for metabolites represented in mmol/L and cell growth in cells/mL. Arrows indicate 50% medium exchange.

Nutrient and metabolite profiles are shown up to day 5 for the Biostat 2 L BR and up to day 7 for the STR50. Glucose consumption and lactate production increased throughout the culture for both the Biostat 2 L and the STR50 BRs (Figure 2A, B). Glucose levels decreased to 9.4 mM by day 5 in the Biostat 2 L and 11.7 mM by day 7 in STR50 cultures while the lactate concentration in the medium reached 31.0 mM by day 5 and 30.1 mM by day 7 for the 2 L and STR50, respectively. Cumulative lactate-to-glucose ratios were compared and found to be near a value of 2, as expected. The Biostat 2 L and STR50 BRs both showed decreasing levels of alanyl-glutamine throughout the culture (Figure 2C, D), with levels dropping to 0.6 mM by day 5 for the Biostat 2 L and 0.1 mM by day 7 for the STR50. Glutamine levels in the Biostat 2 L and the STR50 showed an initial increase in the concentration of glutamine as shown in Figure 2E, F. We postulate that this finding is due to the breakdown of the alanyl-glutamine dipeptide in the culture medium, effectively resulting in a production rate of glutamine that outpaced the consumption rate by the cells, leaving a net increase in glutamine. As the cultures progressed and reached higher cell densities, glutamine consumption increased and glutamine levels ultimately decreased to 0.5 mM by day 5 for the Biostat 2 L and 0.1 mM by day 7 for the STR50. Ammonia levels for the culture are also shown in the figures. Ammonia levels increased for both Biostat 2 L and STR50 cultures over time, with the Biostat 2 L reaching 2.8 mM by day 5 and the STR50 reaching 3.2 mM by day 7.

### 3.5. Critical Quality Attributes of WJMSCs are Maintained After Expansion Under Stirred Conditions in a STR50 Bioreactor

**Figure 3:**
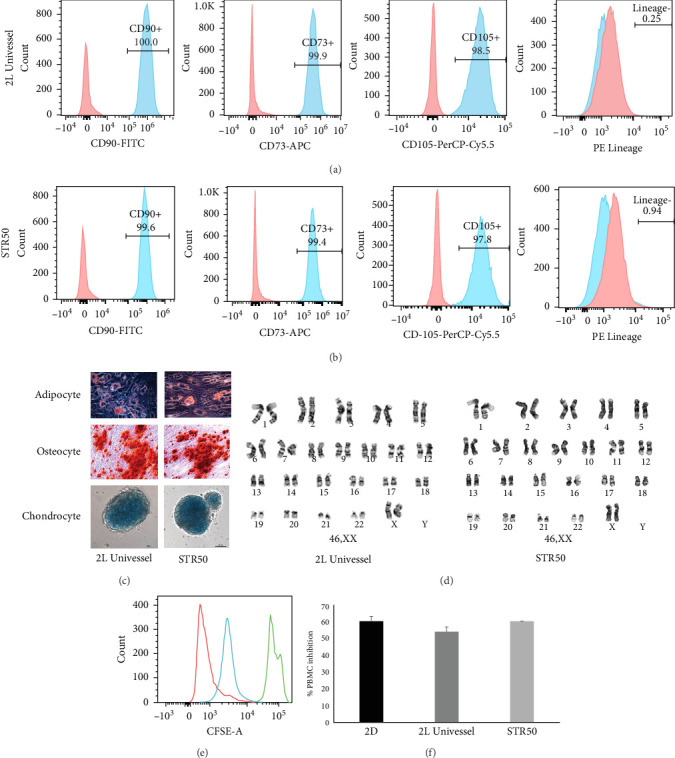
WJMSCs retain CQA after expansion in 2 and 50 L stirred-tank bioreactor. (A) Immunophenotypic analysis of WJMSCs following expansion on microcarriers (10 cm^2^/mL) in 2 L and (B) 50 L bioreactor using xeno-free culture media. Red histograms represent the unstained controls, and the blue solid peak represents the marker indicated. (C) Cells expanded in 2 and 50 L bioreactors maintained their ability to differentiate into adipocytes (oil red O-stained lipid droplets), osteoblasts (Alizarin Red stained calcium deposits), and chondroblasts (alcian blue stained extracellular matrix proteins). Scale bars: 100 μm. (D) 2 and 50 L expanded WJMSCs retain normal karyotype postharvest. (E) Inhibition of PBMCs proliferation in vitro when cocultured with WJMSCs (WJMSC: PBMC-1:10) expanded in traditional 2D flasks, and on microcarriers in 2 and 50 L bioreactors. Representative CFSE histograms of PHA-activated PBMCs (green on day 0), PHA-activated PBMC cultured with 3D-expanded WJMSCs (blue on day 3), and PHA-activated PBMCs (red alone on day 3). (F) The 2D and 3D (2 L and STR50) bioreactor expanded WJMSCs demonstrating the inhibition of proliferation in PHA-activated PBMCs when cocultured for 3 days. The difference in inhibition between 2D and 3D expanded WJMSCs was not significant.

Following the 3D large-scale expansion of clinical-grade WJMSCs in Biostat 2 L and STR50 BRs, we conducted thorough characterizations to ensure their suitability for clinical applications. The expanded cells were assessed for immunophenotypic markers, differentiation potential, immunosuppressive function, karyotype integrity, and sterility. Comprehensive quality testing confirmed the absence of bacterial, fungal, mycoplasma, and endotoxin contamination, following criteria established in prior Phase I trials [23]. At passage 5, the WJMSCs demonstrated ≥95% positivity for CD73, CD90, and CD105 while expressing ≤2% of negative markers (HLA-DR, CD19, CD45, CD34, CD11b, CD14, and CD79) (Figure 3A, B). They retained adipogenic capacity (lipid droplet formation), osteogenic differentiation (extracellular calcium deposits visualized with alizarin red), and chondrogenic differentiation (proteoglycan deposition), meeting ISCT's minimal criteria for MSCs (Figure 3C) [24]. Further, karyotypic analysis showed no chromosomal abnormalities in WJMSCs harvested from the 3D BR culture (Figure 3D). Functional assays demonstrated that both 2D and 3D expanded WJMSCs effectively suppressed lymphocyte proliferation in coculture with PHA-activated PBMCs, achieving inhibition rates above the 20% threshold set by our clinical-grade acceptance criteria (Figure 3E, F). These results align with the requirements for our established acceptance criteria for clinical grade WJMSCs manufactured for an expanded Phase Ib clinical trial in IND#017672 [23] (NCT03158896) (Table 3). This comprehensive evaluation fulfilled the final criterion for a successful dynamic 3D expansion process that we had outlined, that is, the effective large-scale harvest efficiency of WJMSCs from the MC and adherence to regulatory standards.

## 4. Discussion

The large-scale expansion of MSCs is critical to ensure the production of clinically meaningful quantities of viable and functional cells for therapeutic use. Compared to conventional 2D monolayer systems, 3D suspension cultures offer a more efficient and cost-effective alternative by minimizing manual intervention and streamlining expansion workflows [9]. To align with regulatory guidelines, it is essential to develop robust and reproducible bioprocesses using defined components. Additionally, comprehensive characterization of both the process and final cell product is necessary to confirm that the therapeutic cells preserve key functional attributes that are critical for the treatment of the intended disease target. Herein, we developed a process for scaling up WJMSCs to produce a meaningful number of clinical-grade cell doses using a 3D MC-based STR culture system. To our knowledge, the largest WJMSC expansion in STR reported to date was carried out in a 3 L scale with a mean harvest total cell yield of 9.48 × 10^8^ ± 1.07 × 10^7^ cells after 6 days of culture [17]. Our approach involved establishing a seed train starting from 2D monolayer cultures and progressing through MC-based cultivation, and scaling up into controlled 2 and 50 L STRs with optimized culture parameters. Donor variability is a recognized challenge in clinical-grade MSC manufacturing, as differences in donor biology may influence process outcomes and cell product quality. In our previous study, we evaluated multiple WJMSC donors under identical culture conditions on 3D collagen MCs and observed consistent performance across donors, indicating that expansion outcomes were not donor dependent [22]. Likewise, several 2 L runs performed with WJMSCs from independent donors showed no measurable differences in growth kinetics or harvest efficiency, suggesting that trends established at small scale are reproducible in larger stirred-tank formats.

Bioreactor process development and characterization is performed before implementing any new MC-based cell culture system because conditions developed at small scale must translate to large scale. Our prior published work demonstrated the effectiveness of collagen as a MC in promoting WJMSC proliferation under serum-free and xeno-free conditions where various culture parameters were optimized in spinner flask systems [22]. The first characterization step in this study was to identify the critical impeller speed that would promote the just-suspended state (Njs) of the MCs. The Njs should be reached because a productive MC-based culture is typically successful if performed with enough agitation to completely suspend the MCs off the tank bottom without imparting excessive stress onto attached cells. Below Njs, the total MC surface area is not completely or efficiently utilized and cell-MC aggregation can't occur. Therefore, it is important to determine the impeller agitation speed, Njs, at which the Njs is achieved. Agitation affects two critical attributes of the culture: shear force, which should be minimized to avoid cell damage due to small eddy currents, and mixing, which is maximized to ensure even nutrient, cell, and gas distribution. Before culture initiation, mixing studies at the Biostat 2 L and STR50 scales were performed with MCs at 10 cm^2^/mL to empirically identify the minimum agitation rate required to reach Njs in each BR (data not shown). An aeration strategy was also employed to maximize gas exchange and minimize shear forces. This was accomplished by utilizing gas overlay or headspace aeration as the primary method for controlling pH levels and DO concentration. The advantage of implementing this strategy is that cells experience a low-shear environment without foaming, compared to aeration via sparging. This approach provides sufficient control to meet the demand at the cell densities reached in typical MSC cultures. Sparging served as a secondary control method if oxygen demand outpaced the overlay method. In these studies, the overlay control method was sufficient to satisfy oxygen demand, thus it was not necessary to switch to sparge control. The appropriate gassing requirements were controlled utilizing the Biobrain Automation Platform and Software available with the Biostat STR systems. A DO set point of 50% of atmospheric oxygen was chosen for the cultures. It has been found that atmospheric oxygen levels can result in a hyperoxic environment for cultured cells, decreasing proliferation rate and declining metabolic activity [25, 26]. This phenomenon is potentially mediated by the generation of reactive oxygen species [26, 27]. Additionally, in the case of stem cells, hyperoxia has been reported to promote differentiation and change responses to growth factors [28].

The culture parameters described above and outlined in Table 1 were implemented in the Biostat 2 L and STR 50 (30 L working volume). The BR process we developed in serum/xeno-free conditions resulted in a harvest density of approximately 1.24 x10^6^cells/mL in a STR50 at the end of culture period at day 7. This cell density is significantly higher than reported by other WJMSC expansion studies conducted at small scale (800 mL–3 L) in various MC based STR culture which reported cell densities ranging from 9.9 x 10^4^ to 4.7 x 10^5^ cells/mL [17, 19].

Alternative BR platforms—including wave, hollow fiber, and fixed-bed systems—have been evaluated for MSC expansion but remain limited for large-scale WJMSC manufacturing. Wave BRs provide a gentle and low-shear environment but are not optimized for MC-based adherent culture, and the rocking motion can generate shear forces at the liquid–film interface that risk cell detachment. Hollow-fiber systems offer large surface area and perfusion-based nutrient exchange and have been successfully used for high-yield extracellular vesicle/exosome production; however, they frequently develop mass-transfer gradients and present technical challenges for efficient bulk cell harvest at production scale. Fixed-bed systems can achieve high local cell densities with reduced bulk shear, but their packed geometry can give rise to nutrient and oxygen gradients and complicate uniform cell recovery as scale increases. Stirred-tank reactors offer homogeneous mixing, tight control of pH, DO, temperature and agitation, and straightforward scalability by adjusting working volume and MC concentration, which supports cGMP-compliant and large-scale MSC manufacturing. However, STRs require careful optimization to mitigate shear stress, maintain uniform MC suspension, and enable reproducible and high-efficiency downstream harvesting without compromising cell quality—important considerations when translating to clinical scale.

Evaluating the quality and functional integrity of WJMSCs postexpansion in 3D suspension culture systems is critical for clinical manufacturing. In this study, WJMSCs were expanded using MC-based cultures in both 2 and 50 L stirred BRs, followed by a thorough postharvest analysis. Key release parameters—such as viability, phenotypic marker expression, sterility, genomic stability, and both in vitro and in vivo potency—were assessed and compared to quality benchmarks established for 2D-expanded clinical products. Our results demonstrated that the cells harvested from the 3D system were free from microbial contamination and maintained high expression levels of defining MSC surface antigens, including CD73, CD90, and CD105. Unlike reports indicating a decline in CD105 expression after enzymatic detachment from MCs—often attributed to prolonged enzyme exposure or mechanical agitation—our approach preserved CD105 expression above 95%. This was achieved using a 30 min treatment with 50% TrypLE Select, which yielded a harvest efficiency of 95%, indicating gentle yet effective cell recovery. Importantly, WJMSCs expanded at scale retained their multipotent differentiation capabilities and demonstrated robust immunomodulatory function in lymphocyte proliferation assays. Cytogenetic analysis confirmed chromosomal integrity, supporting the process's suitability for generating high-quality, clinical-grade WJMSCs using scalable 3D culture methods.

In our published study [22], we compared 2D TF expansion with 3D spinner flask–based MC expansion and demonstrated a substantial reduction in cost of goods (CoGs), with TF expansion estimated at $47,550 per patient batch (4 × 10^9^ cells) versus $11,822 for spinner flasks. These findings underscored how improved media utilization, reduced consumables, and lower labor inputs make 3D approaches more cost-efficient, while also enabling significantly greater fold expansion (~27-fold) compared with conventional 2D methods (~6–7-fold). Although the present study focuses on 30 L STR expansion, the same cost-driving principles apply. STRs, while requiring higher upfront capital, offer economies of scale by combining larger batch yields with closed-system processing, thereby lowering the effective cost per million/billion cells compared with smaller-scale systems. In this study, we achieved ~ 27-fold expansion in the 30 L STR, demonstrating that a single batch can generate doses sufficient for multiple patients in the allogeneic setting. STRs offer homogeneous mixing, scalability via MC concentration, and robust process monitoring and control, making them especially well-suited for cGMP-compliant WJMSC manufacturing. Furthermore, the estimated cost of media, reagents, and consumables in our 30 L stirred-tank process is less than USD 5000 per billion cells—a level of efficiency not practically achievable with planar culture systems. The scalability of 3D MC-based suspension culture also enables production of clinically relevant doses with fewer expansion runs, improving throughput and reducing facility and labor requirements. For academic settings, smaller-scale platforms may remain practical for early-phase trials; however, for commercial manufacturing, large-scale BR processes—supported by automation and optimized downstream workflows—will be essential to ensure cost efficiency, batch-to-batch consistency, and broad clinical translation.

Thus, the combination of the BR culture system with serum-free and xeno-free media presented herein offers a feasible, faster, and more monitored and controlled alternative to 2D static planar cell expansion technologies for large-scale manufacturing of WJMSCs intended for clinical purposes.

## Figures and Tables

**Table 1:** Final harvest results of the WJMSC microcarrier-based expansion, including total cells, % viability, and fold increase for the 17-day process.

Results from the expansion of cells from 2D culture to the 3D STR50 bioreactor						
	CellSTACK5 (0.424 L)		Univessel (2 L)		STR50 (30 L)	
	Seed	Harvest	Seed	Harvest	Seed	Harvest
Cells/mL	3.3e4	3.3e5	4.5e4	1.1e6	4.5e4	1.24e6
Total cells	1.4e7	1.4e8	9.0e7	2.2e9	1.35e9	3.72e10
Viability (%)	94.2	97.9	97.9	91.0	91.0	93.7
Fold increase	—	10x	—	24.4x	—	27.6x

**Table 2:** Summary of the process flow with operating parameters and bioreactor set points for the run.

Cell bank		1 x CellSTACK5		Biostat (2 L)		STR50 (50 L)
WJMSC	⟶	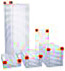	⟶	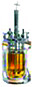	⟶	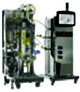
Microcarrier type		n/a		Collagen		Collagen
Seeding density (cells/cm^2^)		4.5e3		4.5e3		4.5e3
Surface area per mL (cm^2^/mL)		7.5		10		10
Media type		MSC Nutristem XF + 5% PLT		MSC Nutristem XF + 5% PLT		MSC Nutristem XF + 5% PLT
Media (mL)		424		2000		30,000
Total surface area (cm^2^)		3180		20,000		300,000
Mass MC (g)		n/a		55.6		833.3
Total cell # required for seeding		1.4e7		9.0e7		1.4e9
Culture duration (days)		4		6		7
BR parameters and set points		n/a		Temp: 37°C pH: 7.35 via CO_2_ (overlay) and 0.5N NaOH DO: 50% via overlay stir speed: 55 RPM		Temp: 37°C pH: 7.35 via CO_2_ (overlay) and 0.5N NaOH DO: 50% via overlay stir speed: 30 RPM
Maintenance		None		1 x 50% media exchange, day 4		1 x 50% media exchange, day 4
Harvest enzyme		0.5 x TrypLE		0.5 x TrypLE		0.5 x TrypLE

**Table 3:** Quality control parameters tested for clinical grade manufactured WJMSCs expanded in a single-use STR50 (50 L stirred-tank bioreactor).

Analytical test	Method	Acceptance criteria	WJMSCs expanded in 50 L
Appearance	Visual	Off-white, opaque, and homogeneous suspension following mixing	Off-white, opaque, and homogeneous suspension, no clumps
Identity	Flow cytometry	≥95% CD73, CD90, CD105 ≤2% CD45, CD34, CD11b, CD19, and HLA DR (lineage negative)	99.6% CD90, 99.4% CD73, 97.8% CD105 ≤1% lineage negative
Purity	Flow cytometry	≥80%	90%
Cell viability	Flow cytometry	≥80%	95%
Potency by immunosuppression	Inhibition of PHA-induced proliferation of PBMCs by WJMSCs	≥20%	60.4%
Endotoxin	Bacterial endotoxin USP <85>	≤5 EU/mL	≤0.05 EU/mL
Mycoplasma	Biochemical luminescence assay	Absence of mycoplasma	Negative
Sterility	Microbiological control of cellular product USP <71>	Absence of micro organisms	Sterile
Chromosomal stability	Karyotype	Absence of chromosomal alterations	No chromosomal abnormalities

## Data Availability

All data supporting the findings of this study are available within the article. Any request for further information should be directed to the corresponding author.
